# Deep leaning-based ultra-fast stair detection

**DOI:** 10.1038/s41598-022-20667-w

**Published:** 2022-09-27

**Authors:** Chen Wang, Zhongcai Pei, Shuang Qiu, Zhiyong Tang

**Affiliations:** School of Automation Science and Electrical Engineering, Beihang University, Beijing, 100191 China

**Keywords:** Computer science, Software

## Abstract

Staircases are some of the most common building structures in urban environments. Stair detection is an important task for various applications, including the environmental perception of exoskeleton robots, humanoid robots, and rescue robots and the navigation of visually impaired people. Most existing stair detection algorithms have difficulty dealing with the diversity of stair structure materials, extreme light and serious occlusion. Inspired by human perception, we propose an end-to-end method based on deep learning. Specifically, we treat the process of stair line detection as a multitask involving coarse-grained semantic segmentation and object detection. The input images are divided into cells, and a simple neural network is used to judge whether each cell contains stair lines. For cells containing stair lines, the locations of the stair lines relative to each cell are regressed. Extensive experiments on our dataset show that our method can achieve 81.49$\%$ accuracy, 81.91$\%$ recall and 12.48 ms runtime, and our method has higher performance in terms of both speed and accuracy than previous methods. A lightweight version can even achieve 300+ frames per second with the same resolution.

## Introduction

With a long research history in computer vision, stair detection is a fundamental problem and has a wide range of applications. Two kinds of mainstream methods are available for stair detection: line extraction methods^1–4^ and plane extraction methods^5–7^. For the first type of method, the staircase is defined as a collection of parallel lines. Lines are extracted by applying Canny edge detection, Hough transform and other traditional computer vision algorithms in RGB images or depth images. For the second type of method, the staircase is defined as a collection of parallel planes in three-dimensional space, and the planes are extracted by applying a plane segmentation algorithm to the point cloud data.

Line extraction methods mainly include traditional computer vision methods and deep learning methods. The main idea of traditional methods is to use visual clues obtained through edge detection algorithms such as the Canny algorithm, the Sobel filter and line detection algorithms such as the Hough transform^8–10^. After performing line extraction, the features of the stairs need to be matched. Khaliluzzaman et al.^11^ regard the endpoints of the stairs as three line segments converging at one point for feature extraction. Carbonara and Guaragnella^12^ creatively regard the stair structure as a periodic signal in the spatial domain, and its period is the distance between two continuous edges. Then, the 2D fast Fourier transform (FFT) is applied to transform the observed signal to the frequency domain to obtain an image that contains only the edges of stairs. Khaliluzzaman et al.^13^ propose a framework based on a unique geometrical feature of a stair. The unique geometrical feature is that every step’s height gradually decreases from the bottom to the top of the staircase. Huang and Tang^14^ propose a method to identify stairs by using the statistical properties of projection histogram. Relatively few deep learning computer vision methods are available for stair detection. The main idea of these methods is to extract the region of interest (ROI) containing stairs in the input image through object detection algorithms such as Yolo^15^ and RCNN^16^. Then, the traditional computer vision methods are applied to extract lines in the ROI^17,18^. These methods divide the stair detection task into two steps, which makes it difficult to ensure real-time performance. In addition, some classification methods utilize deep learning to determine whether an image contains stairs and whether the ROI is upstairs or downstairs^19^. Such a classification method does not achieve pixel-level stair localization. It can only be used to provide voice reminders for visually impaired people and not for robot environment perception.

The main idea of plane extraction method is to extract potential planes from the input point clouds and filter the planes belonging to stairs in a certain way. Point cloud segmentation is a common plane extraction method, and many methods have been developed for stair feature matching. Classifying planes by obtaining their normal vector and eliminating the planes that do not belong to the stairs is a common method^20,21^. Sinha et al.^22^ present an algorithm for stair detection from point clouds based on a new minimal 3D map representation and the estimation of step-like features that are grouped based on adjacency in order to emerge dominant staircase structures. Oh and Choi^23^ propose a stair plane extraction algorithm based on supervoxel clustering. Tang et al.^24^ use the random sample consensus (RANSAC) algorithm to extract planes and then model the corresponding stairs. Stahlschmidt et al.^25^ obtain the ground plane through the analysis and processing of point clouds and then detect a group of continuous rising planes as stair features.

These two types of methods have long provided relatively reliable stair detection abilities for robots used in urban environments and for visually impaired people. However, there are still some important and challenging problems to be addressed.

The working environment of stair detection determines that a related algorithm usually runs on some small embedded devices. This requires an extremely low computational cost to achieve better real-time performance. To solve this problem, the method used by most algorithms is reducing the input data, namely, reducing the size of the input image or the number of three-dimensional point clouds. For example, Shahrabadi et al.^1^ directly discard the regions without stairs in the input images by a priori ROI. Oh and Choi^23^ directly remove large planes to reduce the number of input point clouds when scanning the environment.

The working nature of stair detection determines that a related algorithm should have high reliability and accuracy. The most challenging problem with stair detection is the adaptability of an algorithm to deal with extreme lighting conditions, special structures, severe occlusions and special materials, as shown in Fig. . For the method based on line extraction, these problems will be fatal. The reason for this is that the limitations of traditional computer vision based artificial feature extraction make the algorithms difficult to adapt to complex and changeable scenarios. For the method based on plane extraction, the acquisition of point clouds depends on light detection and ranging (LiDAR) or depth cameras, which are not affected by stair texture features and lighting conditions. However, LiDAR and binocular sensors are often expensive and still cannot solve the problem of severe occlusion.

**Figure 1:**
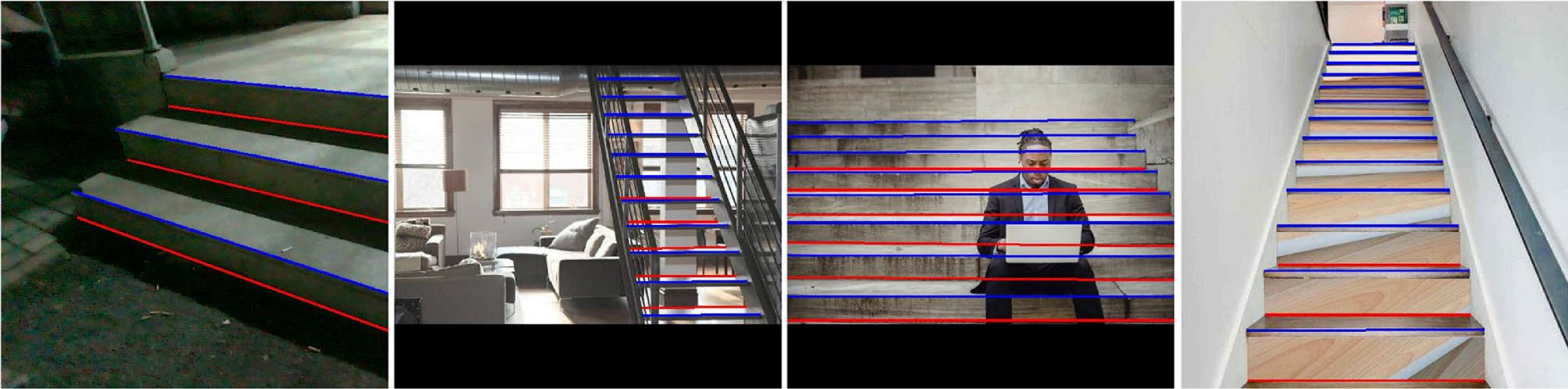
Illustration of the difficulties encountered in stair detection. The convex lines are marked with blue, and the concave lines are marked with red. The figure shows some challenging scenarios, including extreme lighting conditions, special structures, severe occlusions and special materials.

In addition, when detecting objects with texture features and structures that are similar to stairs, these objects are often misidentified as stairs. The reason for this is that an algorithm based on feature extraction cannot obtain high-level semantic information about stairs like humans, which often leads to false detection and missed detection.

With the above motivations, we propose an end-to-end method based on deep learning that has an extremely fast speed and solves the problem regarding adaptability to different scenarios with monocular vision. Since AlexNet^26^ established the dominant position of convolutional neural networks (CNNs) in computer vision in 2012, CNNs have rapidly developed in various fields of computer vision due to their strong learning abilities and unique perception modes. The reason for introducing CNNs into stair detection is that an artificial neural network can learn the texture features and high-level semantic information of stairs simultaneously and obtain better robustness by learning dataset that contain various detection scenarios.

Our method is also based on line extraction. The network input is a RGB image obtained from visual sensor, and the output is a group of extracted stair lines. All the intermediate processes are computed within the neural network. The key problem of designing this neural network is the representation of staircase features. Approximately two schemes are available for this purpose. (1) The stair detection task is regarded as a semantic segmentation task. The pixels belonging to the stair lines can be taken as positive samples, and the background pixels can be taken as negative samples. However, because the stair lines are usually very thin, the numbers of positive and negative samples will be seriously unbalanced, and the network may have difficulty converging. Additionally, the semantic segmentation framework usually incurs a high computational cost. (2) The stair detection task is regarded as an object detection task, and each stair line is given an external rectangular box. It is easy to know that most boxes will be narrow and long. After experiments, we find that the object detection network has difficulty learning the features of stair lines.

**Figure 2:**
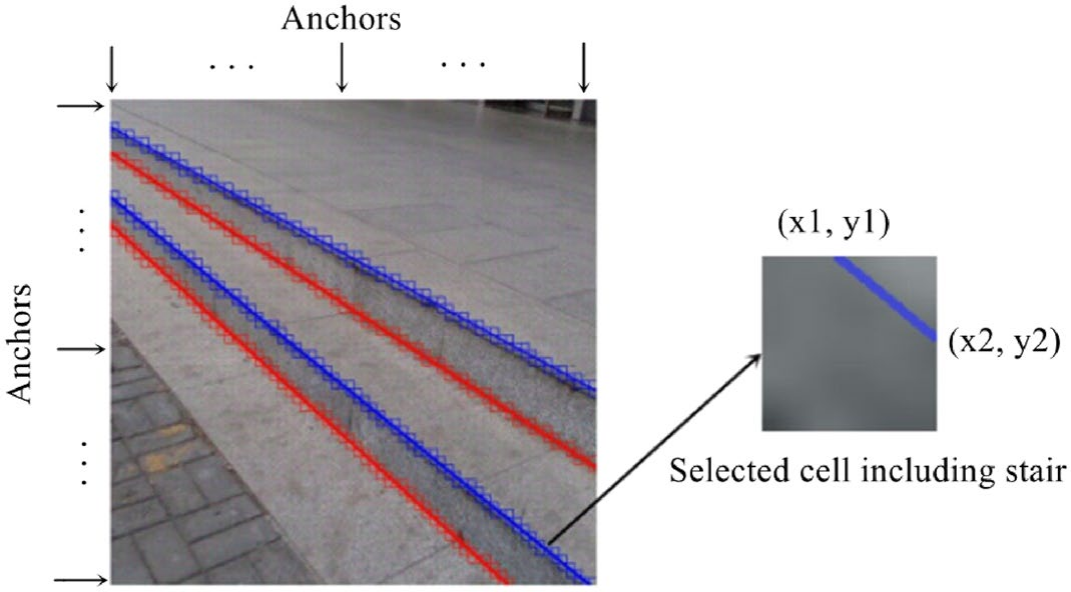
Illustration of our stair feature representation. The whole image on the left is divided into 64 $\times$ 64 anchors, and the anchors (including stair lines) are detected on the right. The normalized coordinates of the two endpoints relative to the upper left corner of the anchor are regressed.

After comprehensively considering these two schemes, we propose a feature representation method of coarse-grained semantic segmentation combined with object detection, as shown in Fig. 2, which can solve the imbalance between positive and negative samples in Scheme 1 and the narrow and long boxes in Scheme 2. As the algorithm is based on coarse-grained segmentation, the size of the final output feature map is 64 $\times$ 64, which greatly reduces the computational cost relative to that of the traditional semantic segmentation network. Specifically, we divide the whole input image (input size: 512 $\times$ 512) into 64 $\times$ 64 small cells which have a size of 8 $\times$ 8, and a feature map with a size of 64 $\times$ 64 is obtained through three downsampling operations. Then, the network is divided into two branches. One is used for classification to judge whether the cell contains convex lines and whether the cell contains concave lines, and the other is used for location. We regard the cells as the anchors of object detection, and in each anchor, the normalized coordinates of the stair line relative to the upper left corner of the anchor are regressed. The two branches work together to detect the whole staircase. In addition, we expand the receptive field by applying dilated convolution and atrous spatial pyramid pooling (ASPP)^27^ in the network to improve its perception ability in scenarios without visual clues, such as scenarios with occlusion and scenarios with extreme lighting.

In summary, the contribution of this work can be summarized in three parts: (1) we provide a stair dataset with fine annotations for stair detection research. The training set contains 2670 images, and the validation set contains 424 images. Each label contains the locations of the two endpoints and the classification (convex/concave) of each stair line. (2) We propose a novel stair detection method based on line extraction. To the best of our knowledge, this is the first deep learning-based end-to-end stair detection network (called StairNet). Compared with the line extraction method based on traditional computer vision, our approach not only achieves extremely fast detection speed but also solves the problem regarding the difficulty of detecting staircases in challenging scenarios. (3) We design a module based on dilated convolution and group convolution. Specifically, we build an inception^28^ module by applying group dilated convolution with different dilation rates in the horizontal and vertical directions, and a channel attention mechanism is also applied so that the network can learn to extract long-range information features with different aspect ratios.

## Methods

In this section, we describe the details of our method, including the network architecture of StairNet and the group dilated convolution with different dilation rates in the horizontal and vertical directions. Finally, we briefly introduce the design of the employed loss function.

### Network architecture

**Figure 3:**
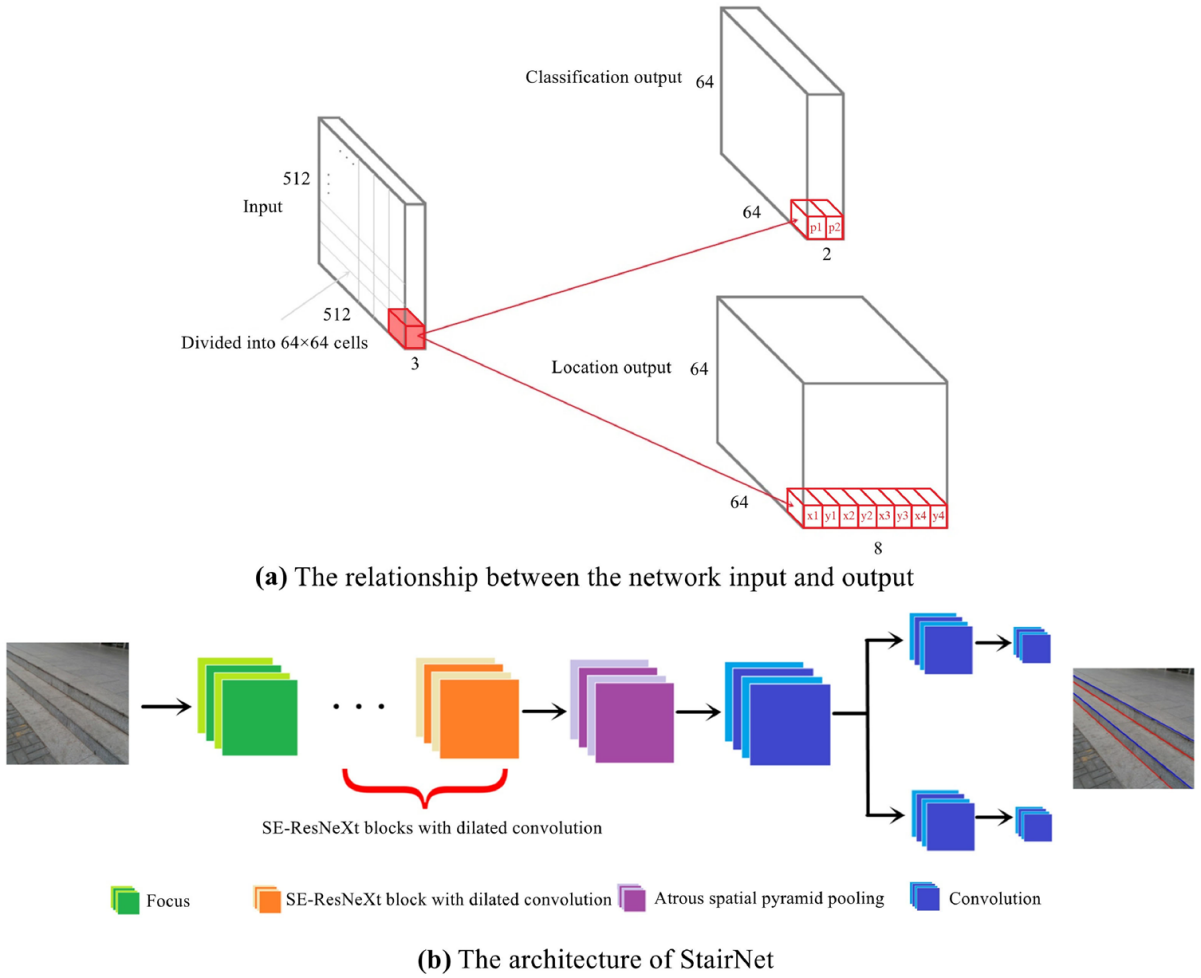
Illustration of the network architecture. (**a**) The relationship between the network input and output. The network takes a 512 $\times$ 512 image as input, and the output of the network is divided into two branches. The output target values for the classification branch are stored in a 3D tensor of size 64 $\times$ 64 $\times$ 2, which is used to judge whether the cell contains convex lines and whether the cell contains concave lines. The output target values for the location branch are stored in a 3D tensor of size 64 $\times$ 64 $\times$ 8, which is used to predict the locations of two sets of stair lines. (**b**) The architecture of our network. The backbone contains a focus module, several SE-ResNeXt blocks with dilated convolution and an ASPP module.

As described above, we propose a feature representation method involving coarse-grained semantic segmentation combined with object detection for stair detection. Our model takes a 512 $\times$ 512 full-color image as input and processes it with a fully convolutional architecture. A feature map with a size of 64 $\times$ 64 is obtained after three downsampling operations. The output of the network is divided into two branches, and each cell location in the 3D output tensor is associated with a multidimensional vector, as shown in Fig. 3a.

The output target values for the classification branch are stored in a 3D tensor of size 64 $\times$ 64 $\times$ 2. Considering that a cell may contain both convex lines and concave lines, we use two independent logical classifiers instead of the softmax function^29^ to judge whether the cell contains convex lines and whether the cell contains concave lines.

The output target values for the location branch are stored in a 3D tensor of size 64 $\times$ 64 $\times$ 8, and each cell predicts two sets of locations (x1, y1, x2, y2) and (x3, y3, x4, y4). Regardless of the posture of the stair line in the cell, (x1, y1) and (x3, y3) always represent the location of the left endpoints, and (x2, y2) and (x4, y4) always represent the location of the right endpoints. In addition, the reason for the prediction of two sets of locations is that a few cells will contain two stair lines after image segmentation. For cells with only one stair line, the two sets of locations are given the same values.

As shown in Fig. 3b, the backbone network consists of a focus module^30^, several squeeze-and-excitation (SE)-ResNeXt blocks^31,32^ with dilated convolution and an ASPP module. Each part is introduced in detail below:

#### Focus module

In the shallow calculation process of a deep neural network, due to the large size of the input image, downsampling is usually required. Common downsampling methods include pooling, convolution with stride >1 and tensor slicing. Pooling causes the loss of details due to the reduction in resolution, which is unfavorable for segmentation^33^. A convolution with stride >1 can extract features while downsampling. Tensor slicing takes a value every other pixel in the feature map and four feature maps are obtained, then the divided feature maps are concatenated together and the number of channels becomes four times of the original layers. Finally a 1 $\times$ 1 convolution is applied to change the number of channels. This operation retains the original features as much as possible while downsampling. For stair detection, we believe that the texture features of stairs should be kept in the shallow layers of the network. Therefore, we use a focus module as the initial module of the network for downsampling. The focus module is essentially a tensor slicing operation, which is similar to the pass-through layer in Yolov2^34^.

#### SE-ResNeXt block

Based on the ResNeXt block^32^, we add the channel attention mechanism of SENet and apply dilated convolution to obtain larger receptive field. ResNeXt^32^ uses standard group convolution in its bottlenecks, and the group convolution can reduce the computational cost and achieve higher accuracy than ResNet^35^. For the details of our bottleneck, see Section “Dilated group convolution with different dilation rates”. Inspired by ENet^36^, standard SE-ResNeXt blocks and SE-ResNeXt blocks with dilated convolution are connected in series to form our backbone. See Table 1 for the detailed architecture of the StairNet.

**Table 1:** StairNet architecture.

Name		Type		Output size	
Initial		Tensor slice		256 $\times$ 256 $\times$ 64	
Bottleneck 1.0		Downsampling		128 $\times$ 128 $\times$ 256	
Bottleneck 1.1				128 $\times$ 128 $\times$ 256	
Bottleneck 1.2				128 $\times$ 128 $\times$ 256	
Bottleneck 2.0		Downsampling		64 $\times$ 64 $\times$ 512	
Bottleneck 2.1		Dilated (1,2) and (2,2)		64 $\times$ 64 $\times$ 512	
Bottleneck 2.2				64 $\times$ 64 $\times$ 512	
Bottleneck 2.3		Dilated (2,4) and (4,4)		64 $\times$ 64 $\times$ 512	
Bottleneck 2.4				64 $\times$ 64 $\times$ 512	
Bottleneck 2.5		Dilated (3,8) and (8,8)		64 $\times$ 64 $\times$ 512	
Bottleneck 2.6				64 $\times$ 64 $\times$ 512	
Bottleneck 2.7		Dilated (4,16) and (16,16)		64 $\times$ 64 $\times$ 512	
**Repeat bottlenecks 2.0 to 2.7 without downsampling operation of bottleneck 2.0**					
ASPP				64 $\times$ 64 $\times$ 512	
Conv 3 $\times$ 3				64 $\times$ 64 $\times$ 128	
classification	location	classification	location	classification	location
Conv 3 $\times$ 3	Conv 3 $\times$ 3			64 $\times$ 64 $\times$ 128	64 $\times$ 64 $\times$ 128
Conv 1 $\times$ 1	Conv 1 $\times$ 1			64 $\times$ 64 $\times$ 2	64 $\times$ 64 $\times$ 8
	Sigmoid		Activation	64 $\times$ 64 $\times$ 2	64 $\times$ 64 $\times$ 8

The bottlenecks with downsampling have a stride = 2 in the 3 $\times$ 3 convolution and the bottlenecks without downsampling have a stride = 1 in the 3 $\times$ 3 convolution.

**Figure 4:**
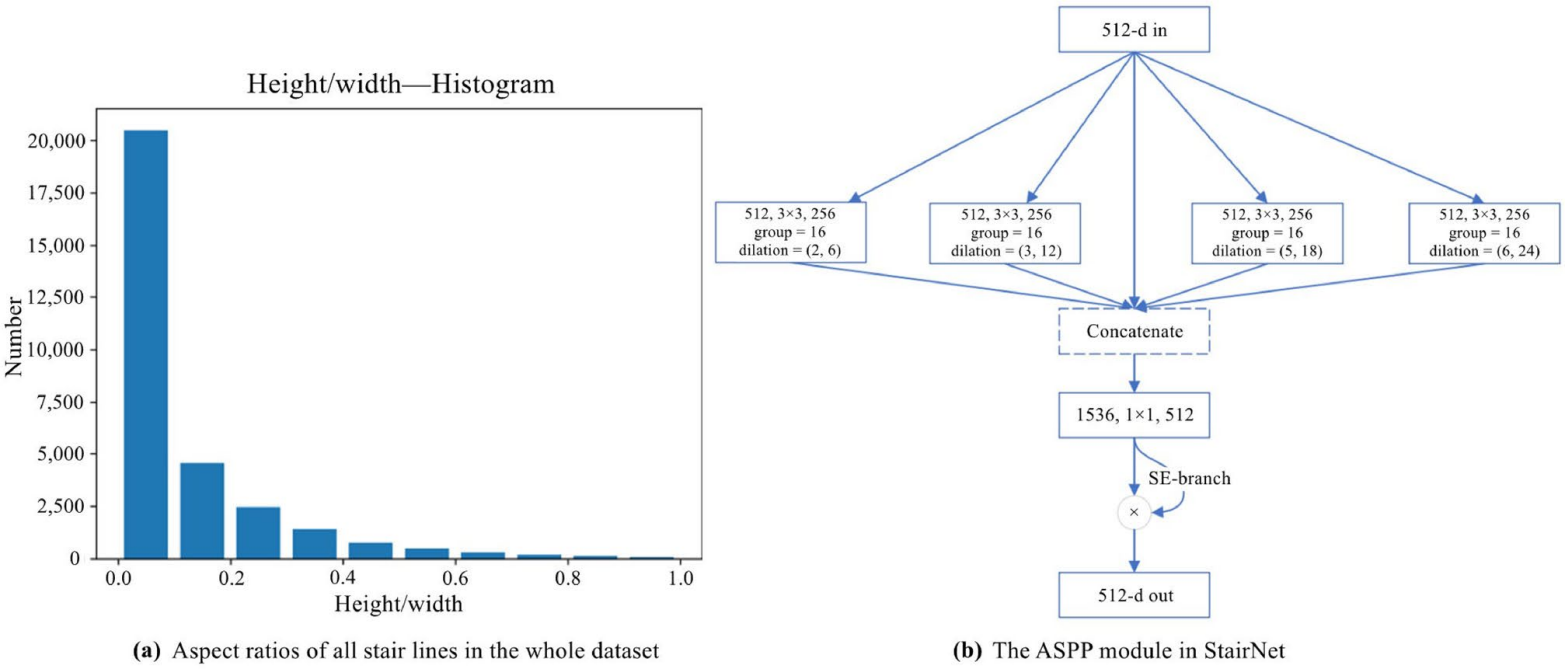
(**a**) The aspect ratios of all stair lines in the whole dataset. The aspect ratios of most stair lines are within the range of 0–0.2. (**b**) The ASPP module in StairNet: Our ASPP module has five branches, including an input feature map and group dilated convolutions with dilation rates of (2,6), (3,12), (5,18) and (6,24). At the end of the module, a channel attention mechanism is added to learn the emphasis placed on context information extraction at different scales. A layer is denoted as (input channels, filter size, output channels).

#### ASPP module

To further expand the receptive field and enhance the learning ability of our network with respect to semantic stair information, we apply an ASPP module. Reference^27^ applies dilated convolution with dilation rates of (6,6), (12,12), (18,18) and (24,24) to extract features in parallel to capture object and image context information at multiple scales. We apply a module similar to the ASPP module and replace the dilation rates with (2,6), (3,12), (5,18) and (6,24). The reason for applying dilated convolution with different dilation rates in the horizontal and vertical directions is that the distributions of stair lines in the dataset are usually transverse. Therefore, dilated convolution with a larger transverse dilation rate is conducive to the detection of stair lines. Specifically, we count the aspect ratios of all stair lines in the whole dataset, and the histogram is shown in Fig. 4a. The aspect ratios of most stair lines are within the range of 0–0.2, so we set the aspect ratios of dilation rates at approximately 0.2. In addition, our ASPP module contains a parallel branch for the input feature map and adds a channel attention mechanism to learn the emphasis placed on context information extraction at different scales, as shown in Fig. 4b.

**Figure 5:**
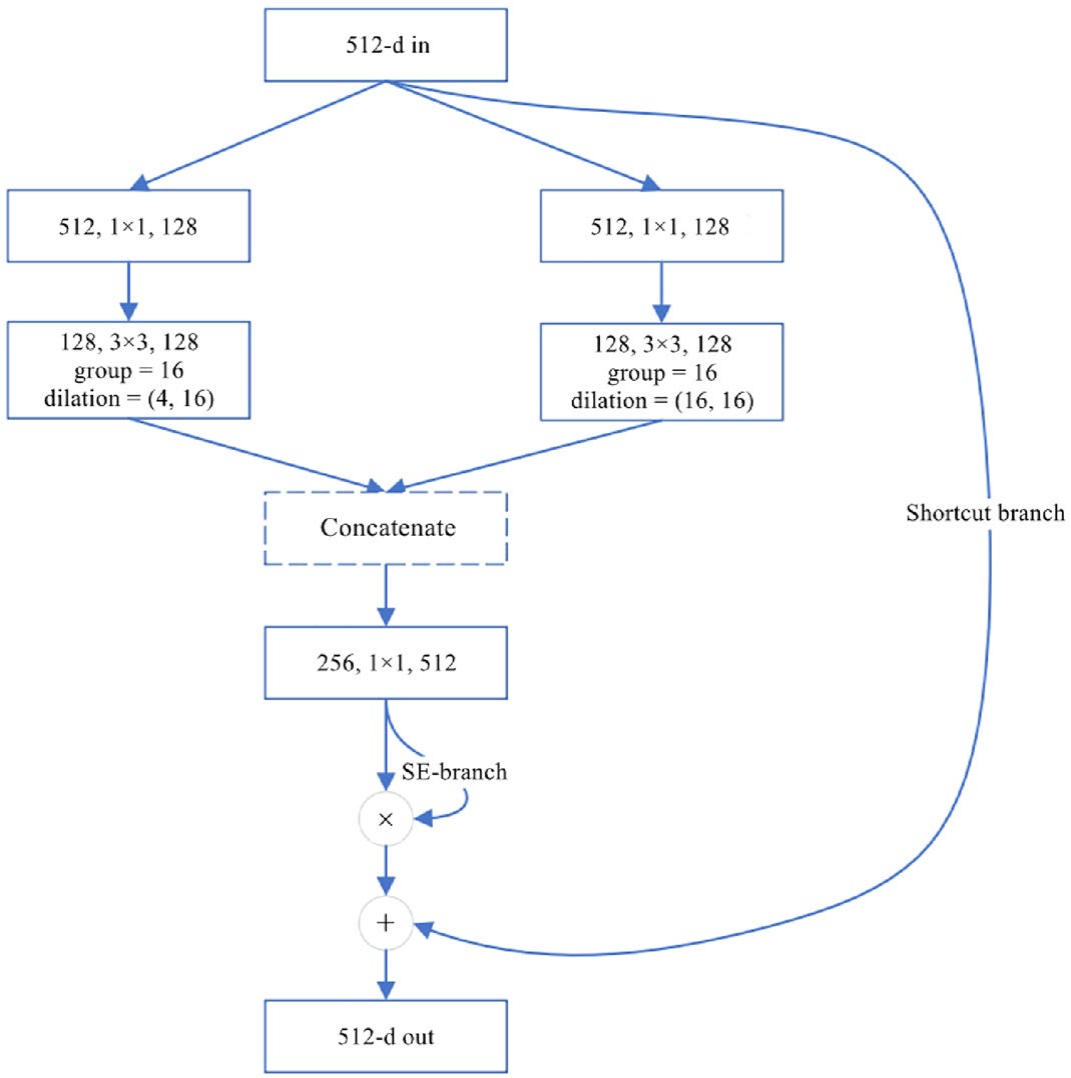
Illustration of the SE-ResNeXt block with dilated convolution. The original standard group convolution with 32 groups is divided into two branches with 16 groups, and the dilation rates of the dilated convolutions are (4,16) and (16,16). Finally, the results of the two branches are concatenated. A layer is denoted as (input channels, filter size, output channels).

### Dilated group convolution with different dilation rates

As mentioned in “SE-ResNeXt block”, to increase the receptive field and improve the segmentation performance of our network, we apply dilated convolution in the SE-ResNeXt block. While applying dilated convolution, we redesign the standard group convolution of the ResNeXt block. We build an inception module by applying group dilated convolutions with different dilation rates in the horizontal and vertical directions. Specifically, we concatenate the calculation results of standard dilated convolutions and the calculation results of dilated convolutions with different dilation rates in the horizontal and vertical directions, and then we apply a channel attention mechanism to learn the weights of the concatenated channels. Figure 5 shows the structure of Bottleneck 2.7 in Table 1, and other bottlenecks can be obtained in the same way.

Similar to the dilation rates in the ASPP module in “ASPP module”, we consider that the distributions of stairs in the dataset are usually transverse. To use this prior knowledge, we calculate asymmetric dilated convolutions with dilation rates of (1,2), (2,4), (3,8), and (4,16) and their corresponding standard dilated convolutions and then concatenate the results. An asymmetric dilated convolution is helpful for learning the features of a single stair line, while the standard dilated convolution is helpful for learning the contextual features between stair lines.

### Loss function

Stair detection is a typical multitask of classification and regression. Our loss function inherits the multitask loss idea used for most object detection tasks. The loss function includes a classification loss and a location loss. The specific formula is as follows:

1 $$\begin{aligned} \begin{aligned}{}&L(\{p_{ij}\},\{t_{ij}\})=\frac{1}{N^{2}}\sum _{i}^{N}\sum _{j}^{N}(L_{cls}(p_{ij},p_{ij}^*)+\lambda p_{ij}L_{loc}(t_{ij},t_{ij}^*)) \end{aligned} \end{aligned}$$

where N represents the number of cells in a row or column, namely, 64, and i and j represent the position of the cell in the whole image. $p_{ij}$ is a 2-dimensional vector that indicates the prediction probability regarding whether the cell contains convex lines and whether the cell contains concave lines. The corresponding ground truth $p_{ij}^*$ has four values: (1,0), (1,1), (0,1) and (0,0), which represent only convex lines, both convex lines and concave lines, only concave lines and no lines, respectively. $t_{ij}$ is an 8-dimensional vector, which represents the normalized coordinates of the two sets of locations predicted by the cell. $t_{ij}^*$ is the corresponding ground truth. $\lambda$ is the weight coefficient, which is set to 4 here. Since we only calculate cells that contain stair lines, we use the vector $p_{ij}$ dot vector $L_{loc}$ with the broadcasting mechanism of PyTorch.

For the classification loss $L_{cls}$, the binary cross-entropy loss function with sigmoid activation is applied to judge whether the cell contains lines. For the location loss $L_{loc}$, the mean square loss function is applied. According to the prior knowledge that stairs are usually distributed horizontally in an image, we need to strengthen the localization of the ordinate. Therefore, the location loss is divided into two parts according to the abscissa and ordinate, and these parts are given different weights, that is:

2 $$\begin{aligned} L_{loc}(t_{ij},t_{ij}^*)=L_{loc}(x_{ij},x_{ij}^*)+\alpha L_{loc}(y_{ij},y_{ij}^*) \end{aligned}$$

where $x_{ij}$ is a 4-dimensional vector that represents the 4 abscissa values predicted by the cell and $x_{ij}^*$ is the corresponding ground truth. $y_{ij}$ is a 4-dimensional vector that represents the 4 ordinate values predicted by the cell, and $y_{ij}^*$ is the corresponding ground truth. $\alpha$ is the weight coefficient, which is set to 4 here.

## Results

In this section, we describe the details of the conducted experiment, including the introduction of the dataset, the model evaluation method, the training strategy, ablation experiments, performance experiments and comparison experiments with traditional methods on the dataset.

### Experimental settings

This section includes the following three parts: the introduction of our dataset, the training strategy and the evaluation metrics we designed.

#### Dataset introduction

**Figure 6:**
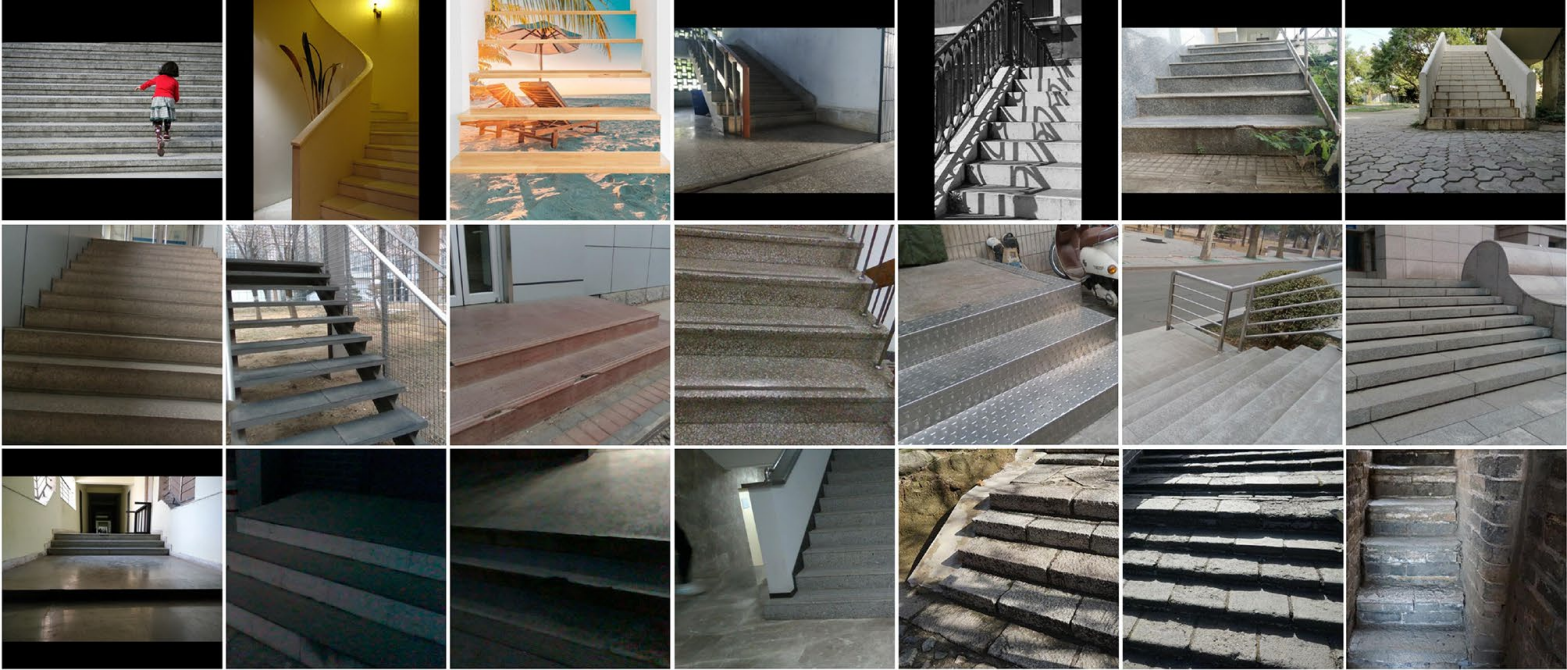
Partial images found in the training set.

The images in the dataset are collected from actual scenes, and all of them are padded and resized to 512 $\times$ 512 to simplify the data loading process. The whole dataset contains a total of 3094 images, which are randomly divided into 2670 images for the training set and 424 images for the validation set. Figure 6 shows some images in the training set.

The annotation form of the dataset is as follows:

cls x1 y1 x2 y2/n

...

Each stair line is represented by the above five-tuple data, where cls represents the class of the stair line, 0 represents a convex line and 1 represents a concave line. X1 and y1 represent the coordinates of the left endpoint of the stair line, and x2 and y2 represent the coordinates of the right endpoint of the stair line. The coordinate system in our dataset uses the pixel coordinate system and the coordinate origin is located in the upper left corner of the image, with the x direction pointing to the right and y direction pointing down. The label of an image is stored in a text file and associated by the file name.

#### Training strategy

We train the model on a workstation with an i7-9700 CPU and an RTX 3080 GPU by using the PyTorch framework. As mentioned above, the input size of the network is 512 $\times$ 512, training is conducted for a total of 200 epochs, and the batch size is set to 4. The Adam optimizer is used, the weight decay is set to $10^{-6}$, and the initial learning rate is set to 0.0005. In addition, we apply a dynamic learning rate adjustment strategy, where the learning rate is halved every 50 epochs.

In terms of data enhancement, we mainly use a random mirror with a probability of 0.5 and random occlusion with a probability of 0.5. Random mirror is used to eliminate the uneven distribution of ROIs in the training images. Random occlusion is used to simulate the situation in which the stairs are often blocked by pedestrians and other objects in reality.

#### Evaluation metrics

In essence, our task is still an object detection problem based on coarse-grained segmentation. There are only four kinds of cells in our task, namely, cells with only convex lines, cells with only concave lines, cells with both kinds of lines and cells with no lines. Since the background cells are easy to classify and a few cells with both lines are difficult to locate, to objectively evaluate the performance of the model, we use the frequency weighted intersection over union (FWIOU)^37^ as the evaluation method, and the background class is not calculated.

3 $$\begin{aligned} FWIOU=\frac{1}{\sum \nolimits _{i=0}^{k}\sum \nolimits _{j=0}^{k}p_{ij}}\sum _{i=0}^{k}\frac{\sum \nolimits _{j=0}^{k}p_{ij}p_{ii}}{\sum \nolimits _{j=0}^{k}p_{ij}+\sum \nolimits _{j=0}^{k}p_{ji}-p_{ii}} \end{aligned}$$

where $p_{ij}$ is the number of pixels of class i inferred to belong to class j. Namely, $p_{ii}$ represents the number of true positives (TP), while $p_{ij}$ and $p_{ji}$ are usually interpreted as false positives (FP) and false negatives (FN), respectively. Then, the above formula can be rewritten as follows:

4 $$\begin{aligned} FWIOU=\frac{1}{\sum \nolimits _{i=0}^{k}\sum \nolimits _{j=0}^{k}p_{ij}}\sum _{i=0}^{k}\frac{\sum \nolimits _{j=0}^{k}p_{ij}TP}{TP+FP+FN} \end{aligned}$$

When applying Eq. (4), $p_{ij}$ is regarded as the number of cells of class i inferred to belong to class j. The judgment of true positives and false positives depends not only on the classes of cells but also on the locations of lines in these cells. In other words, a TP cell must meet the following two conditions: (1) the cell is a positive sample and is correctly predicted as a positive sample. (2) In the cell, the location error between the predicted location of the line and the corresponding ground truth is within a certain threshold.

In the object detection task, the intersection over union(IOU) is used to measure the proximity between two boxes. Similarly, we need an index to measure the proximity of two line segments. The location of a line segment is determined by its two endpoints, so the problem can be transformed into measuring the proximity between endpoints. Inspired by reference^38^, we apply Eq. (5) to convert the distance between two endpoints into a confidence score.

5 $$\begin{aligned} c(x)= {\left\{ \begin{array}{ll} \frac{e^{\alpha \left( 1-\frac{D_T(x)}{d_{th}}\right) }-1}{e^{\alpha }-1}, &{} if\,D_T(x)\le d_{th} \\ 0,&{} \text {otherwise} \end{array}\right. } \end{aligned}$$

where c(x) is the confidence and $D_T(x)$ is defined as the 2D Euclidean distance in the image space. $d_{th}$ is the distance threshold and is set to 1. The sharpness of the exponential function is defined by the parameter $\alpha$. To achieve precise localization with this function, $\alpha$ is set to 2. In practice, since a line segment has no direction, we apply Eq. (5) to an endpoint on one line segment and the endpoint closest to it on the other line segment. After obtaining the confidence of the two endpoints, we calculate the mean value and assign it as the final confidence score.

In the following content, we use the accuracy, recall and FWIOU metrics when c(x) = 0.5, as well as the mean FWIOU (mFWIOU), as the evaluation indicators of the model. The mFWIOU is defined by the mean value of the FWIOUs obtained under 19 confidence values when c(x) = 0.05–0.95 with a step size of 0.05.

### Ablation experiments

In this section, we verify our method with several ablation studies. The experiments are all conducted with the same settings as those described above. We take the network stacked with standard SE-ResNeXt blocks as the baseline. Based on this, we study the influence of the focus module, ASPP module and SE-ResNeXt blocks with dilated convolution on the performance of the model. The experimental results are shown in Table 2.

**Table 2:** Results of ablation experiments.

Backbone	Dilation	Focus	ASPP	Accuracy (%)	Recall (%)	FWIOU (%)	mFWIOU (%)
SE-ResNeXt (baseline)				79.96	81.41	67.83	58.13
SE-ResNeXt + dilation	$\checkmark$			81.07	81.92	69.00	59.11
SE-ResNeXt + focus		$\checkmark$		80.56	80.90	67.91	58.07
SE-ResNeXt + ASPP			$\checkmark$	81.27	80.93	68.45	58.57
SE-ResNeXt + dilation + focus	$\checkmark$	$\checkmark$		81.47	81.68	69.11	59.32
SE-ResNeXt + focus + ASPP		$\checkmark$	$\checkmark$	81.24	81.32	68.69	58.79
SE-ResNeXt + dilation + ASPP	$\checkmark$		$\checkmark$	81.47	81.13	68.71	59.06
StairNet	$\checkmark$	$\checkmark$	$\checkmark$	81.49	81.91	69.31	59.51

The results show that the dilated convolution can significantly improve the performance of the model. In addition, the focus module and ASPP module slightly improve the performance of the model.

### Performance experiments

In this section, we present model performance experiments conducted on the validation set, which are mainly performed to determine the inference speed and accuracy of the model. We provide three versions of StairNet, including StairNet 1$\times$, StairNet 0.5$\times$ and StairNet 0.25$\times$, to meet the requirements of devices with different computation capabilities. The size of the model is scaled by a channel width factor which is directly multiplied by the number of channels in the network. We test the three versions on a desktop platform and an embedded platform. The specific experimental platforms, model sizes and inference speeds are shown in Table 3.

**Table 3:** Results of the model inference speed experiments.

Platform	StairNet 1$\times$(35.1 mb) (ms)	StairNet 0.5$\times$(9.1 mb) (ms)	StairNet 0.25$\times$(2.48 mb) (ms)
i7-9700 + RTX 3080	12.48	5.92	3.07
NVIDIA Jetson NX	219.07	97.45	42.31

The results show that our three models can meet the real-time requirements of the desktop platform, and the 0.5$\times$ and 0.25$\times$ models can meet the real-time requirements of the embedded platform.

To objectively evaluate the accuracy of the model, we divide the data in the validation set into daytime dataset, night dataset and art dataset according to their collection conditions, and the detection difficulty also increases in sequence. The accuracy results are shown in Table 4.

**Table 4:** Results of the model accuracy experiments.

Model	Accuracy (%)			Recall (%)			FWIOU (%)			mFWIOU (%)		
	Day	Night	Art	Day	Night	Art	Day	Night	Art	Day	Night	Art
StairNet 1$\times$	86.29	79.24	72.21	87.05	83.43	68.94	76.65	68.61	54.97	66.00	58.91	46.78
StairNet 0.5$\times$	85.32	78.81	71.00	86.55	83.29	68.53	75.53	68.17	53.97	64.78	57.79	45.71
StairNet 0.25$\times$	82.84	75.52	67.80	84.73	81.20	66.96	72.28	64.38	51.21	62.03	55.17	43.40

The results show that the performance of the 0.5$\times$ model is slightly worse than that of the 1$\times$ model, but the performance of the 0.25$\times$ model is much lower than that of the 0.5$\times$ and 1$\times$ models. For all models, the accuracy on the daytime dataset is greater than the accuracy on the night dataset and the art dataset.

**Figure 7:**
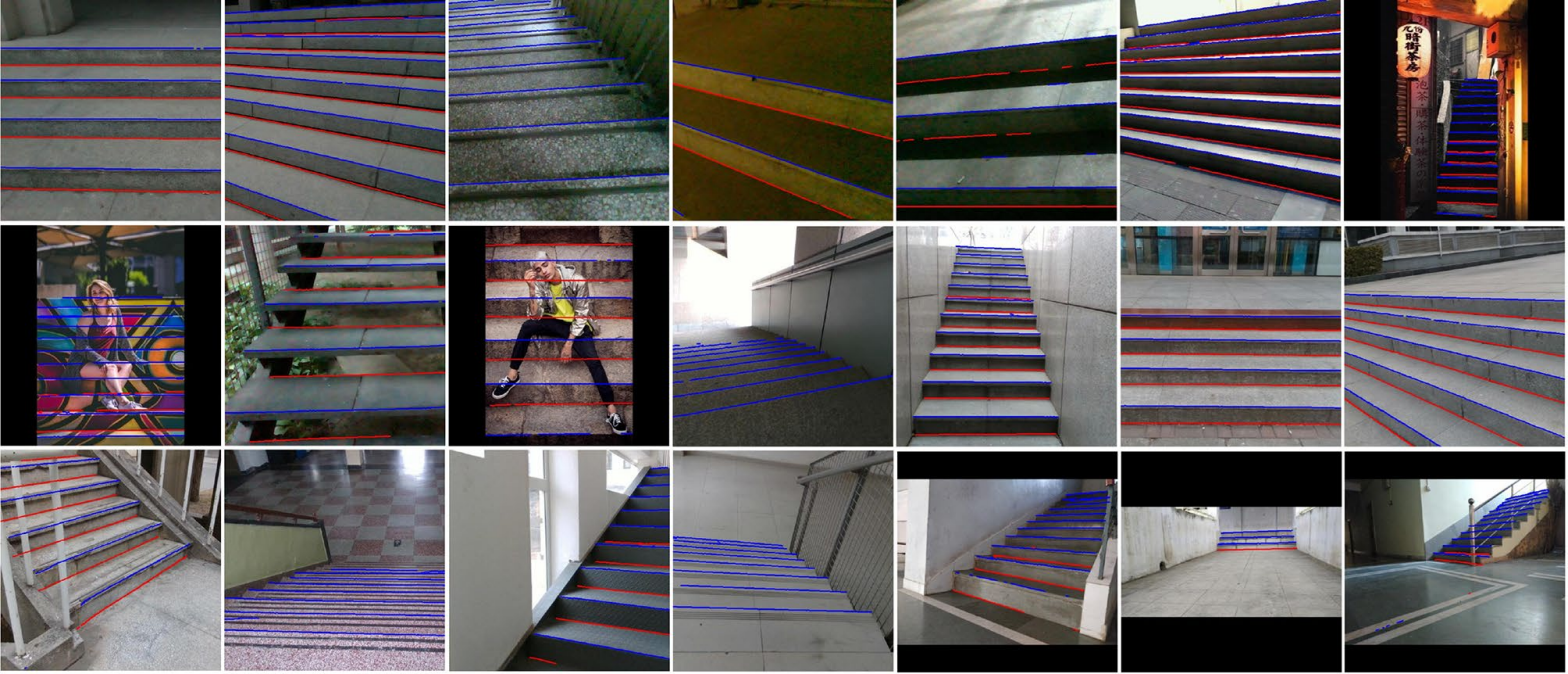
Partial visualization results produced by StairNet 1$\times$ on the validation set.

Figure 7 shows some visualization results produced by StairNet 1$\times$ on the validation set. These staircases have different building structures, materials, shooting angles and lighting conditions. Our method can still obtain satisfactory results under the conditions of extreme lighting, serious occlusion and special stair structures and materials.

### Comparison experiments with traditional methods

In this section, we compare our method with traditional methods. For the method based on traditional computer vision^8–10^, we apply Gabor filter, Canny algorithm and Hough transform to make our implementation. First, we use Gabor filter to enhance the features in the 90$^{\circ }$ direction, because stairs are usually distributed horizontally in the images. Then the Canny algorithm is used for edge detection, and a binary image with edge information is obtained. Finally, we use Hough transform to find all the potential lines in the binary image. For the deep learning method combined with traditional computer vision^17,18^, we apply YoLov5^30^ to obtain the ROI containing stairs, and then the traditional computer vision algorithms are applied to extract lines in the ROI.

Before the performance evaluation, we adjusted the parameters of Gabor filter, Canny algorithm and Hough transform to fit our validation set. After the evaluation, we convert the outputs of the above two methods to matrix outputs that are consistent with StairNet. In addition, because StairNet can distinguish the convex lines and the concave lines but the above two methods cannot, we convert the classification output of StairNet into two classes: cells with lines and cells without lines. The comparison experiments are conducted on our validation set, using the evaluation metrics described in “Evaluation metrics” and the same platform described in “Training strategy”. The results are shown in Table 5.

**Table 5:** Results of the comparison experiments.

Method	Accuracy (%)	Recall (%)	Runtime (ms)
Gabor + Canny + Hough (Our implementation)	21.2	33.6	10.67
YoLov5 + Gabor + Canny + Hough (Our implementation)	37.3	34.1	28.19
StairNet 1$\times$-Ours	82.03	83.07	12.48
StairNet 0.5$\times$-Ours	80.85	82.24	5.92
StairNet 0.25$\times$-Ours	78.81	81.62	3.07

**Figure 8:**
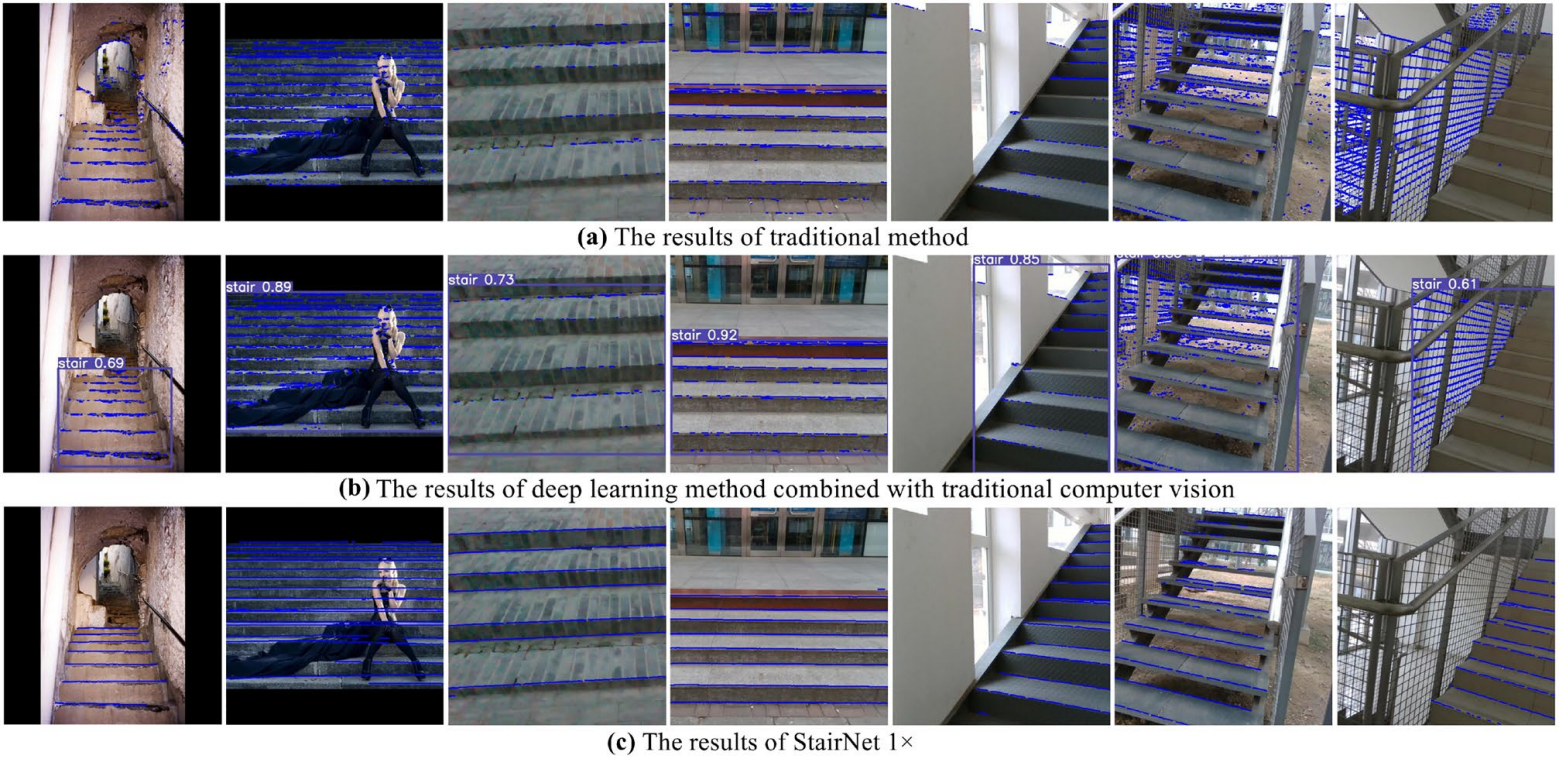
Partial visualization results produced by traditional method, deep learning method combined with traditional computer vision and StairNet 1$\times$ on the validation set. Our StairNet performs much better than traditional method and deep learning method combined with traditional computer vision in the challenging scenarios. It is also obvious from the figure that our StairNet has better accuracy and better recall.

The results show that the performance of the StairNet is far superior to the traditional methods in terms of accuracy and recall. Our validation set contains a variety of scenarios and our evaluation metrics are very strict, so it is difficult to find a set of parameters to adapt to a variety of detection scenarios for traditional methods. For the inference speed, our StairNet 1$\times$ is slightly slower than the method based on traditional computer vision. However, our StairNet 0.5$\times$ and StairNet 0.25$\times$ are much faster than the traditional methods. In addition, due to the data transfer between CPU and GPU, the deep learning method combined with traditional computer vision is the slowest. Figure 8 shows some visualization results produced by traditional method, deep learning method combined with traditional computer vision and StairNet 1$\times$ on the validation set.

## Discussion

We propose a novel fully convolutional neural network architecture for stair detection. First, considering the slender shape of stair lines, we creatively propose a new feature representation. We regard the stair detection task as a combination of semantic segmentation and object detection. The input images are divided into cells, a simple neural network is used to judge whether each cell contains stair lines and regress the locations. In the ablation experiments, the baseline network has an accuracy of 79.96%, which proves the effectiveness of our proposed feature representation method. Next, according to some prior knowledge of the stair lines, we propose several optimization methods, including: (1) the texture features of stairs should be learned in the shallow layers of the network. Therefore, we use a focus module as the initial module of the network. (2) Because stair lines are typical long-range information features and are often distributed horizontally in the images, we use the dilation convolution and ASPP module to expand the receptive field while reducing the number of downsampling. And we set different dilation rates in the horizontal and vertical directions to make the network learn from different scales and directions. In the ablation experiments, the effectiveness of our proposed optimization methods are proved. Then, we compress the original model and conduct performance experiments on the models. The results show that the accuracy decreases slightly as the model is compressed, but the inference speed is greatly improved. The experiments of 0.5$\times$ and 0.25$\times$ models on Jetson NX prove the real-time performance on the embedded system, which provide an edge computing solution for stair detection in various devices. Finally, we compare our method with traditional methods on our validation set. The results show that the performance of our StairNet is far superior to the traditional methods in terms of accuracy and recall, and the 0.5$\times$ and 0.25$\times$ models are also much faster than the traditional methods.

There have been some previous studies based on line extraction methods. Most of them use traditional computer vision algorithms, such as Canny edge detection, Hough transform, etc. Because stairs have well described geometric features, and there is not enough large-scale stair dataset for deep learning training in the industry, the detection methods based on traditional computer vision have been used as a means of stair detection for a long time. Although, these algorithms often run on CPU and have good real-time performance on the host end^8^, it is not possible to migrate to the embedded system. In order to overcome the poor adaptability of traditional computer vision algorithms under challenging scenarios, including extreme lighting conditions, special structures, severe occlusions and special materials, there are some improvements. For example, some studies first extract the ROI containing stairs in the input image through object detection algorithms, and then the traditional computer vision algorithms are applied to extract lines in the ROI^17,18^. This single object detection requires only a small-scale dataset to be trained and reduces the interference of complex scenarios to some extent. However, the detection task is divided into two stages, which are carried out on CPU and GPU respectively, and the real-time performance is poor. In order to fundamentally solve the problems, we propose an end-to-end detection method based on deep learning for the first time, which does not rely on any external algorithm and achieves better real-time performance while improving the detection robustness. This depends on the collection and production of our dataset, which contains a total of 3094 images, and each stair line is labeled at the pixel level according to the novel stair feature representation method we proposed. In the experiments, we find that the dataset of this scale is enough for network convergence and learning.

There are also some methods based on line extraction which extract stair lines from both RGB and depth maps^2,39^. This is due to the maturity and miniaturization of depth cameras today. These methods can extract the stair features in different dimensions, and RGB map and depth map have their own advantages in different scenarios. However, These methods require rules designed artificially to integrate the results of RGB map and depth map. There is still the problem of poor adaptability, and the algorithms need to process two images at the same time, which has higher requirements on the computing power of the device. In this paper, the method of deep learning is used to detect stairs quickly and accurately under the condition of monocular vision. However, the experimental results show that the performance on the night dataset and the performance on the art dataset are lower than the performance on the daytime dataset. The reason is that the information from monocular RGB images is limited, especially at night, it is difficult to distinguish stairs in RGB images. In future research, we will consider combining depth information for stair detection, and study the integration of texture information from RGB map and depth information from depth map in neural network.

## Conclusion

We proposed a novel fully convolutional neural network architecture that regards the stair detection task as a combination of semantic segmentation and object detection, where the aim is to quickly and accurately detect stair lines in monocular vision in an end-to-end manner. In addition, we provide a dataset with fine annotations for stair detection research. Experiments conducted on this dataset demonstrate the effectiveness of our method. Finally, experiments performed on a Jetson NX show that our model can run in real time on an embedded platform and serve as an edge computing solution for stair detection in various devices. In future work, the depth data will also be used for stair detection and the neural network architecture can effectively process RGB-D data to obtain higher accuracy.

## Data Availability

Our dataset is available at https://data.mendeley.com/datasets/3jjdm6rn96/1.
